# The *N*-formyl peptide receptors: much more than chemoattractant receptors. Relevance in health and disease

**DOI:** 10.3389/fimmu.2025.1568629

**Published:** 2025-03-04

**Authors:** Filomena Napolitano, Nunzia Montuori

**Affiliations:** ^1^ Department of Translational Medical Sciences, University of Naples Federico II, Naples, Italy; ^2^ Center for Basic and Clinical Immunology Research (CISI), World Allergy Organization (WAO) Center of Excellence, University of Naples Federico II, Naples, Italy

**Keywords:** chemoattractant receptors, G-protein coupled receptors, *N*-formyl peptide receptors, inflammation, receptor structure-function

## Abstract

Pattern Recognition Receptors (PRRs) are a superfamily of receptors that detect molecular structures typical for pathogens and damaged cells and play a crucial role in the proper function of the innate immune system. A particular subgroup of membrane-bound PRRs is represented by the N-formyl peptide receptors (FPRs) that consist of transmembrane G-protein coupled receptors involved in inflammatory responses. FPRs were initially described in immune cells as transducers of chemotactic signals in phagocytes that react to tissue injury. Subsequently, FPRs were also identified in a wide variety of cell types, including cancer cells. Beyond broad cellular distribution, FPRs are also characterized by the ability to bind a variety of ligands with different chemical and biological properties, ranging from natural peptides to synthetic compounds. The binding of FPRs to specific agonists induces a cascade of functional biological events, such as cell proliferation, migration, angiogenesis, and oxidative stress. From all this evidence, it becomes clear that FPRs are multifaceted receptors involved in several pathophysiological processes associated with inflammation. In this review, we provide a comprehensive molecular description of structure-function relationship of FPRs and their pivotal role in the host defense, highlighting the regulatory functions in both the initiation and resolution of inflammation. In addition to their activity as PRRs during innate immune response, we focus on their involvement in pathological conditions, including chronic inflammatory disease, neurodegenerative disorders, and cancer, with special emphasis on FPR targeting as promising therapeutic strategies in the era of precision medicine.

## Introduction

The innate immune system serves as the first-line defense and provides rapid, non-specific protection against pathogens. It is now accepted that innate immune response is crucial for the development of adaptive immunity and the regulation of diverse physiological processes (1). Critical modulators of innate immune response are Pattern Recognition Receptors (PRRs), a superfamily of receptors that function as nonspecific sensors for pathogen-associated molecular patterns (PAMPs) and/or damage-related molecular patterns (DAMPs) (2). PAMPs are released from pathogens whereas DAMPs are endogenous molecules, including nucleic acids, proteins, ions, glycans, and metabolites, which are released following cell damage and/or stress. The ability of PRRs to recognize both PAMPs and DAMPs allows them to take part in non-sterile and sterile inflammation (3). After DAMP/PAMP recognition, PRRs induce transient pro-inflammatory gene expression and immune cell activation to remove pathogens or restore tissue homeostasis. However, chronic activation of PRR has been linked to chronic inflammation and autoimmune diseases.

Based on their localization, PRRs may be classified into membrane-bound PRRs implicated in the surveillance of extracellular milieu and intracellular PRRs activated by internalized inflammatory stimuli (4). Frequently, the description of PRR sub-families is incomplete as the most prevalent classification in literature includes only Toll-like receptors (TLRs), Nod-like receptors (NLRs), C-type lectin receptors (CLRs), and RIG-like receptors (RLRs) (4). Phagocytic leukocytes also express another class of innate immune receptors, named *N*-formyl peptide receptors (FPRs), which play a key role in host defense and inflammation. To date, three subtypes (FPR1, FPR2, and FPR3) have been identified in humans though the role of FPR2 in inflammation is much debated. They are members of the seven transmembrane receptors, also known as G protein-coupled receptors (GPCRs), which are responsible for transducing a wide range of signals across the plasma membrane (5).

The discovery of FPRs was made possible after the identification and characterization of formylated peptides from which they also take their name. Since prokaryotes, excluding Archaea, initiate protein synthesis with *N*-formyl methionine, chemically synthesized peptides starting with *N*-formyl methionine were used to evaluate neutrophil activation (6, 7). Moreover, formylated peptides are contained in mitochondria from which they are released following tissue and cellular trauma. The data that demonstrated leucocyte migration toward *N*-formyl peptides led to the discovery of FPR1. In early studies, *N*-formyl-methionyl-leucyl-phenylalanine (fMLF) resulted as the most potent agonist for neutrophil chemotaxis (7).

Apart from innate immune response, FPRs are an intriguing PRR class that may have more complex functions than are currently appreciated. This hypothesis is based on two important assumptions: i) FPRs are highly expressed in innate immune cells, but they are also detected on nonhematopoietic cells, including epithelial cells, endothelial cells, hepatocytes and neurons; ii) beyond formylated peptides, high number of FPR ligands eliciting different cellular responses has been reported in the literature. The broad tissue distribution of FPRs corresponds to their wide pathophysiological relevance as demonstrated by several studies ranging from chronic inflammation to cancer. However, the development of FPR-targeting drugs encounters many difficulties due to ligand promiscuity and marked signaling bias downstream of FPRs.

In this review, we discussed the most recent advancements on the biological functions of FPRs, the role of FPRs in the development and progression of inflammatory chronic diseases and how do FPRs function in the tumor microenvironment. This thorough analysis aims to offer a fresh insight into the pathophysiological role of FPRs and establish a sound scientific foundation for future therapeutic approaches.

## Multiple signaling pathways and biological functions of *N*-formyl peptide receptors

In humans, the N-formyl peptide receptor family consists of three isoforms, named FPR1, FPR2, and FPR3, each encoded by a separate gene located on chromosome 19q13 (8). Given that early studies demonstrated that these receptors recognize formylated peptides deriving from bacterial and mitochondrial protein synthesis, FPRs were so named following the criteria of the International Union of Basic and Clinical Pharmacology (IUPHAR) nomenclature of a receptor based on its agonist. Traditionally, FPRs are considered as important mediators of inflammatory and immune responses in pathogen infection and cellular damage though a new scenario is emerging from several studies that reveal new insights on the heterogeneity and the promiscuity of these receptors. In fact, FPRs play a crucial role in pathophysiological processes such as tissue repair and chronic inflammation independently of binding to formylated peptides (9–11).

FPRs are G protein-coupled receptors (GPCRs), also known as seven-(pass)-transmembrane domain receptors containing an extracellular amino terminus, seven transmembrane helices, and an intracellular carboxy terminus. In contrast to the conventional localization of FPRs to the plasma membrane, new paradigms indicate that FPRs also localize to the nucleus, thus participating in intra-nuclear signaling, as demonstrated for FPR2 in lung carcinoma and gastric adenocarcinoma human cell lines (12). Subcellular fractionation has been described also for FPR1 that is stored in primary granules and secretory vesicles of circulating neutrophils (13). Further studies on subcellular fractionation, FPR transport from intracellular compartments to plasma membrane, and receptor cycling are needed.

However, binding of a signaling molecule to FPRs results in G protein activation, which in turn triggers the activation of multiple downstream second messengers. Particularly, FPRs are coupled with inhibitory G subfamily (Gi) of G proteins, as demonstrated by the total loss of cell response to agonists after the exposition to pertussis toxin (PTX) (14). Upon activation, intracellular domain of FPRs mediate signaling to heterotrimeric G-proteins, which dissociate into α and βγ subunits and trigger distinct and divergent signaling pathways. βγ subunit activates phospholipase C (PLC) responsible for calcium mobilization and activation of protein kinases C (PKC), which are crucial for degranulation and superoxide production. βγ subunit also activates phosphoinositide 3-kinase (PI3K)/protein kinase B (Akt) pathway that is necessary for cell chemotaxis and superoxide production. α subunit of G proteins regulates the Ras superfamily of small GTPases, which are critical for cytoskeletal reorganization and lead to the activation of mitogen-activated protein kinase (MAPK) pathways, extracellular-regulated protein kinase 1/2 (ERK1/2), p38, and JUN-N-terminal protein kinase (JNK) (15). Notably, α subunit of G proteins also regulates the assembly and activation of NADPH oxidase, crucial for innate immunity as during phagocytosis it produces superoxide, thus contributing to the elimination of invading microorganisms. Particularly, FPR activation induces reactive oxygen species (ROS) production through GTP bound-Rac1 and ERK 1/2 pathways and the direct binding of Rac1 to the NADPH oxidase cytosolic component p67^phox^ is crucial to form the active oxidase (10).

Since membrane lipid rafts are crucial to organizing the complicated trafficking and signaling of GPCRs, it is conceivable that lipid rafts can serve as platforms for integrating signal transduction processes in FPR signaling as well, but further investigations should be undertaken to explore the exact molecular and biochemical mechanisms. To date, cholesterol-rich plasma membrane rafts have been demonstrated to play a crucial role in intracellular transduction processes in FPR2 signaling (16). Moreover, recent data suggest that the constitutive internalization of FPR3 occurs through a mechanism dependent on caveolae, a distinct subset of lipid rafts enriched in the structural scaffolding protein caveolin (17).

Following activation by ligand, the control and the termination of FPR activities occur through uncoupling from G proteins (desensitization) and receptor internalization, which removes receptors from the cell surface. These mechanisms are crucial to prevent damage to the host in the continued exposure to activating ligand. In general, GPCR desensitization and internalization are mediated by receptor phosphorylation and arrestin binding to the cytosolic domains of the agonist-occupied receptor (18). The internalization of FPRs could occur in the absence of arrestins, which is however required for the recycling of internalized receptor (19, 20). It has been demonstrated that the processes of FPR desensitization and internalization are differentially regulated by phosphorylation at distinct sites within the carboxyl terminus of FPRs (19). In addition to homologous desensitization, FPR activation desensitizes several chemokine receptors, including CXCL8, CXCR4, CCR1, CCR5 by a mechanism defined heterologous desensitization (21–24). The complexity of FPR-mediated signaling pathways is partly linked to their ability to recognize a large variety of pathogen-derived and endogenous ligands and different agonists of the same FPR isoform can trigger distinct signaling cascade. Emerging studies have introduced the concept of “biased agonism” to explain the extensive variation in FPR-mediated signaling pathways. Biased agonism indicates that ligands stabilize the receptors in a specific conformation that triggers one signaling pathway between the different signal transduction pathways associated with the receptor itself. In practice, different agonists specific to an FPR isoform cause distinct effects. Therefore, cellular response is biased towards a specific signaling pathway (25). Of note, the multiple signals transduced by FPRs are also dependent both on ligand concentration and exposure time to ligand (26). Biased agonism dependent on ligand concentration could be determined by the ability of FPRs to express both high and low affinity binding sites for ligands and each binding site could elicit a specific intracellular signaling pathway. Alternatively, different concentrations of ligand and/or exposure time could induce distinct conformational changes in FPRs.

In addition to biased agonism, FPRs can undergo oligomerization that could be responsible for different signaling pathways. It has been reported that FPRs form different higher order structures, including FPR1 homodimers, FPR2 homodimers, FPR2/FPR1 heterodimers, and FPR2/FPR3 heterodimers (27–29). Surprisingly, anti-inflammatory and pro-inflammatory ligands might preferentially act on different FPR oligomers (27). It has been shown that pro-resolving ligands induce the formation of FPR1/FPR2 heterodimers and FPR2 homodimers, whereas pro-inflammatory ligands and FPR antagonists are not able to induce the formation of these structures. Moreover, Cooray et al. demonstrated that FPR2 homodimers might be linked to the activation of the p38/MAPK-activated protein kinase/heat shock protein 27 signaling pathway, exerting immunomodulatory and anti-inflammatory effects, including the release of IL-10 (27). However, the specificity and functional significance of homo- and hetero-dimerization of FPRs has not yet been completely understood. Future studies are needed to provide the molecular mechanism underlying the dual nature of FPRs, especially FPR2, and how agonist binding contributes to receptor dimerization.

The possibility to selectively activate or inhibit specific pathways has attracted molecular pharmacology, thus leading to the development of biased agonists. In fact, a given FPR biased agonist might activate anti-inflammatory and pro-resolving pathways or inhibit Gi protein-mediated proinflammatory activities of the FPRs.

Remarkably, biased agonism-based regulation of FPR signaling and functions represents a valid therapeutic approach for inflammatory diseases. To shed light on the biased signaling mechanisms and favor the development of FPR-biased anti-inflammatory and pro-resolving therapies, conformational studies, crystallography, cryo-EM, and evidence for FPR higher-order structures are needed.

Several functions of FPRs occur through the interaction with the urokinase (uPA) receptor (uPAR). A specific region of uPAR, corresponding to amino acids 88-92 (SRSRY) can interact with FPRs, mediating uPA- or fMLF-dependent cell migration. uPA itself can promote uPAR interaction with FPRs, by determining the exposure of the uPAR_88-92_ region, upon binding to the receptor (30). fMLF-dependent cell migration of epithelial cells requires uPAR interaction with FPR1; on the other hand, uPAR interacts with FPR2 in monocytes and with FPR2 and FPR3 in basophils (9, 31, 32). Recently, it has been demonstrated that uPAR is able to induce cell proliferation in normal skin fibroblasts by interacting with FPR1, FPR2, and FPR3 and the inhibition of a single isoform with specific antibodies causes cell growth arrest (11). A cooperation between the three isoforms is conceivable, most likely through the formation of heterodimers. Moreover, FPRs/uPAR crosstalk induces a proliferative phenotype in skin fibroblasts, by Rac1 and ERK activation, c-Myc Ser62 phosphorylation and Cyclin D1 upregulation that drive cell cycle progression (11). To fulfill the wide range of functions, FPRs belong to a macromolecular complex on cell surface. Cell migration and ROS production require FPR interaction with uPAR and β1 integrin engagement, as demonstrated by experiments performed with P25 peptide, which disrupts uPAR interactions with β1 or β2 integrins (10, 33). A better understanding of the mechanisms by which FPRs interact with β1 integrin - with or without direct interaction - will provide new insights on the biological functions of these receptors.Taken together, these findings suggest that FPRs could modulate both pro- and anti-inflammatory response, depending on the nature and the concentration of the ligand and on the different binding sites of the receptor. The interplay of FPRs with different ligands and how different ligands interacting with the same FPR isoform can trigger opposing effects should be better examined to develop innovative approaches for the treatment of chronic diseases.Interestingly, FPRs are subjected to phenomena of dynamic evolution and neofunctionalization that could partly explain the complexity and promiscuity of these receptors. An early duplication induced FPR1 and FPR2/FPR3 splitting, whereas FPR3 originated from the latest duplication near the origin of primates, as shown by a phylogenetic analysis (34). High frequency of polymorphisms affects FPR expression or function and different responses of FPR haplotypes toward bacterial peptides have been detected (35, 36). Of note, the ligand specificity of FPRs seems to be conserved among species, suggesting a conserved function of FPRs in the immune system (36). Although evolutionary history indicates that FPRs are subjected to purifying selection, ligand-binding sites have experienced positive selection and significant sequence divergence has occurred among mammalian FPR1 genes (34). FPRs are also an example of genetic innovation; genomic events observed in rodents led to expansion and neofunctionalization of FPRs, which have evolved from pathogen sensors in immune cells to olfactory receptors in neurons (37).

Over the past 20 years, it has become increasingly clear that FPRs play a crucial role in host defense, as well as in immune and non-immune inflammatory disorders. Given that FPRs are a complex receptor system, the identity and the contribution of specific isoforms in health and disease remains largely unknown and, certainly, merit further research.

In the next chapter, we aim to report the main data relating to three members of FPRs to shed light on the single contribution of FPR1, FPR2 and FPR3 in pathophysiology through the description of the main ligands activating FPRs. Since the FPR family is known for the structural and biological diversity of their ligands, the multiple FPR ligands discovered over the past years are summarized in **Table 1** and grouped into microbe-derived, endogenous, and synthetic ligands.

**Table 1:** Summary of the main ligands activating FPRs.

Ligands	Selectivity	Functional outputs	Pathophysiologic effects
1) Microbe-derived ligands			
fMLF	FPR1>FPR2	chemotaxis of neutrophils and macrophages, degranulation and superoxide generation	pro-inflammatory
fMAMKKL	FPR1	as above	pro-inflammatory
fMFIYYCK	FPR1	as above	pro-inflammatory
fMKKIML	FPR1	as above	pro-inflammatory
gp41 T20/DP178	FPR1, FPR2	chemotaxis of neutrophils and monocytes, calcium mobilization	pro-inflammatory
gp41 T21/DP1074	FPR1, FPR2	as above	pro-inflammatory
PSMα	FPR2	calcium mobilization, superoxide generation in neutrophils	pro-inflammatory
MCT-2	FPR2	chemotaxis of neutrophils	pro-inflammatory
Hp2-20	FPR2	chemotaxis of monocytes, neutrophils and lymphocytes, gastric mucosal healing	pro-inflammatory/pro-resolving
2) Endogenous ligands			
ANXA1	FPR1, FPR2, FPR3	inhibition of platelet activation and thrombus formation, wound healing	anti-inflammatory/pro-resolving
Ac2-26	FPR1, FPR2	migration of epithelial celland wound healing	anti-inflammatory/pro-resolving
TAFA4	FPR1	chemotaxis of macrophages, phagocytosis, and superoxide generation	pro-inflammatory
LXA4	FPR2	suppresses the release of pro-inflammatory factors, infiltration of immune cells and superoxide generation	anti-inflammatory/pro-resolving
RvD1	FPR2	inhibition of TNF-α and IL-1β, promotion of neutrophil apoptosis and corneal epithelial wound healing	anti-inflammatory/pro-resolving
Aβ42	FPR2	fibrillary formation and brain deposition	neurodegenerative
Humanin	FPR2, FPR3	protection of neuronal cells from apoptosis	neuroprotective
SAA	FPR2	chemotaxis of phagocytes	pro-inflammatory
VIP	FPR2	reduction of pro-inflammatory mediators and neutrophil and macrophage infiltration	anti-inflammatory/pro-resolving
LL-37	FPR2	stimulation of neutrophil functions	pro-inflammatory
F2L	FPR3	calcium mobilization and chemotaxis	pro-inflammatory
Gliadin	FPR1	chemotaxis of neutrophils	pro-inflammatory
suPAR**_88-92_**	FPR1, FPR2, FPR3	chemotaxis of immune cells, degranulation and superoxide generation	pro-inflammatory
3) Synthetic ligands			
WKYMVm	FPR1, FPR2, FPR3	pro-angiogenic, reduction of inflammatory cytokines	anti-inflammatory/pro-resolving
6C-dimethyl-imidazole (1R)-11	FPR2	inhibition of peritonitis-associated neutrophil infiltration	anti-inflammatory
Compound 17b	FPR2	reduction of vascular remodeling associated with diabetes and myocardial infarction	anti-inflammatory/pro-resolving
Compound MR-39	FPR2	reduction of inflammatory process in mouse model of Alzheimer’s Disease	anti-inflammatory
Compound 43	FPR1, FPR2	improvement of infarct structure	anti-inflammatory/pro-resolving
ACT-389949*	FPR2	resolution of cardiac inflammation	anti-inflammatory/pro-resolving
BMS-986235*	FPR2	resolution of cardiac inflammation	anti-inflammatory/pro-resolving
BLXA4*	FPR2	resolution of gingival inflammation	anti-inflammatory/pro-resolving

*Phase 1 clinical trials.

The agonists are grouped into microbe-derived, endogenous and synthetic ligands.

### FPR1 is the first discovered receptor in the FPR family

Among the three FPRs identified in humans, the first detected isoform was FPR1, which is a high-affinity receptor for various short formylpeptides such as the prototypical peptide *N*-formyl-Met-Leu-Phe (fMLF) (38).

The structure of FPR1 consists of 350 amino acids with two putative N-linked glycosylation residues at positions 4 and 10 and a disulfide bond at residues 98↔176 (39). Three putative phosphoserine sites (residues 328, 332, and 338) and four putative phosphothreonine sites (residues 329, 331, 334, 336, and 339) have also been identified (40). The secondary structure consists of seven transmembrane (TM) helixes (TM1 to TM7) characterized by six loops, among which three in the extracellular region and other three in the cytoplasm region (41). The restricted opening at the extracellular surface could explain the higher affinity of FPR1 for short peptides, unlike FPR2 (38).

FPR1 is mainly expressed in myeloid cells, including leukocytes, monocytes, macrophages, natural killer and dendritic cells. It has been also detected in nonhematopoietic cells, such as fibroblasts, epithelial cells, endothelial cells, smooth muscle cells, astrocytes, and hepatocytes (39).

The chemoattractant properties of FPR1 have been widely demonstrated; activated FPR1 plays a key role in the directional migration of phagocytes to the site of bacterial infections (42). During infections, FPR1 is also a crucial modulator of phagocyte degranulation and superoxide generation, by requiring Gi coupling (43).

Distinct FPR1-mediated phagocyte functions are regulated by different concentrations of chemoattractants. It has been demonstrated that FPR1 selectively triggers distinct signaling pathways in human peripheral blood neutrophils based on different concentrations of fMLF; subnanomolar concentrations of fMLF induced chemotaxis and conformational change of FPR1 whereas nanomolar and micromolar concentrations were responsible for degranulation and superoxide generation (44). Of note, the tight regulation of FPR1 by agonist concentration expands our knowledge of innate immunity: first, FPR1 responds with the chemotaxis of phagocytes to avoid damage to not affected tissues; second, it induces bactericidal activities of phagocytes in the site of infection, where the bacterial-derived peptide is at high concentrations.

FPR1 can recognize a variety of formylated and non-formylated, microbe- and host-derived ligands. Beyond the canonical *E. coli*-derived peptide fMLF, FPR1 interacts with other formylated bacterial peptides, including *Salmonella-*derived fMAMKKL peptide, *Staphylococcus*-derived fMFIYYCK peptide, and *Listeria*-derived fMKKIML peptide (45, 46). The microbe-derived non formylated peptides that interact with FPR1 are mainly viral, but further investigations are needed. Particularly, Human Immunodeficiency Virus (HIV) envelope proteins, such as gp41 T20/DP178 and gp41 T21/DP107, have been proposed as ligands of FPR1 though it has been shown that the chemotactic peptides released from HIV-1 gp41 act mainly through FPR2 (47). Both full-length Annexin-1 (ANXA1) and the N-terminal peptide of the protein, Ac2-26, have been proven to interact with FPR1 (48). FPR1 acts as a cytokine ligand, as demonstrated by TAFA4 or FAM19A that represents a novel class of chemokine like ligands involved in the regulation of immune responses within the central nervous system (40, 49). Furthermore, several synthetic low-molecular-weight ligands for FPR1 have been identified and some of these constitute novel promising compounds for the treatment of cancer and other inflammatory disorders (5).

### FPR2 transduces both pro- and anti-inflammatory signals

Among the FPR family, FPR2 is the most versatile and promiscuous isoform both for the variety of structurally diverse ligands and for the ability to mediate also anti-inflammatory and pro-resolving actions. FPR2 is also indicated as ALX/FPR2 since it binds the endogenous ligand A_4_ lipoxin (LXA_4_).

FPR2 is primarily expressed in the membranes of myeloid cells, such as leukocytes, monocytes, macrophages, natural killer and dendritic cells. Non-immune FPR2-expressing cells are mainly epithelial cells, hepatocytes, astrocytoma and neuroblastoma cells, and microvascular endothelial cells (50).

Molecular details regarding the structure and ligand recognition of FPR2 are still under investigation though in the last 5 years numerous efforts have been made to reveal insights into ligand-binding modes of FPR2. To date, there is data on the structure of FPR2 associated only with agonists. It would be useful and interesting to perform studies on FPR2 in an inactive conformation, for example FPR2-bound to specific antagonists. The crystal structure of FPR2 complexed with synthetic peptide WKYMVm showed an active conformation and revealed a canonical seven-transmembrane helical bundle conformation. The extracellular region is composed of an N terminus and three extracellular loops, with β-hairpin conformation of second extracellular loop that is connected to helix III by a disulfide bridge (51). A recent study of the structure of FPR2-Gi complex revealed three important insights into ligand-recognition and signaling: i) a vast space in ligand-binding pocket could explain the ability to recognize long peptides and large proteins; ii) the high flexibility of extracellular region of FPR2 could be linked to the ability to recognize chemically diverse agonists; iii) the amphiphilic nature of ligand-binding pocket fit with the ability to bind pro-resolving lipid mediators (52). Notably, protein and lipid ligands bind FPR2 in distinct binding sites (28).

How FPR2 displays opposing activities has been studied by different approaches. Cooray et al. proposed an unusual molecular mechanism that governs the dual function of FPR2 by regulating pro- and anti-inflammatory activities. Agonist binding and dimerization state determine FPR2 functional response; particularly, anti-inflammatory and pro-resolving signals activate homodimers of FPR2 leading to the release of IL-10, as well as FPR2/FPR1 heterodimers (27). Instead, Zhang et al. proposed the allosteric modulation of FPR2 as molecular mechanism underlying signaling bias abilities. Of note, the preincubation with agonists with pro- and anti-inflammatory effects at very low concentrations elicited different conformational changes in FPR2 (28).

The main feature of FPR2 is represented by the wide variety of endogenous and exogenous ligands including proteins, lipids and various stimulatory mediators. Formylated peptides, including fMLF, bind to FPR1 with a higher binding affinity, as described above, though alpha-type phenol-soluble modulins (PSMα) produced by *Staphylococcus aureus* and mitochondria-derived mitocryptide-2 (MCT-2) prefer to bind to FPR2 (50). *Helicobacter pylori*-derived non-formylated peptide, abbreviated to Hp2-20, is a potent FPR2 agonist and this interaction plays a critical role in the pathological processes associated with *Helicobacter pylori* infection (46). On the other hand, Hp2-20 promotes gastric mucosal healing by interacting with FPR2, thus suggesting a pro-resolving action (53). Furthermore, FPR2 can recognize several host-derived peptides. Among the evidence that indicates the role of FPR2 in facilitating the resolution of inflammation, there is the binding to LXA_4_. In fact, LXA_4_, which derives from arachidonic acid, exerts strong anti-inflammatory and pro-resolving effects (54), but further studies are needed to determine the functional role of LXA_4_ as FPR2 agonist. FPR2 can also act as a pro-resolving mediator by binding to Resolvin D1 (RvD1), which has been proven to exert good regulatory effects on inflammation (55). Apart from the ability to mediate the pro-resolving signal of lipidic mediators, FPR2 binds diverse peptides involved in inflammatory disorders, especially neuroinflammatory disease. Of interest, FPR2 serves as a receptor for β-amyloid peptide (Aβ42), highlighting its involvement in the pathogenesis of Alzheimer’s disease (AD). In contrast to this data, FPR2 has been shown to mediate the neuroprotective effects of humanin (56). This evidence, once again, showed the multifaceted nature of FPR2.

The dual role of FPR2 also emerges from studies on the Serum Amyloid A (SAA) as an agonist for FPR2. SAA is considered as a major acute-phase protein and is involved in the chemotaxis of phagocytes in inflamed tissues. Further studies showed that SAA plays a crucial role in epithelial wound healing to be indicated as an epithelial pro-restitutive factor by Hinrichs BH et al. (57). Thus, SAA could mediate opposing effects via FPR2.

Vasoactive Intestinal Peptide (VIP) is another host-derived peptide that activates FPR2 and directs FPR2 signaling towards the resolution of inflammation (58), whereas LL-37 elicits the proinflammatory action of FPR2 (50).

It is important to underline that the ligands of FPR2 with anti-inflammatory and pro-resolutive action are under investigation to develop new therapeutic strategies for diverse inflammatory conditions. Furthermore, the two opposing roles of FPR2 are still under study and can be relevant in the context of chronic diseases.

New knowledge of the pro-resolving signaling pathways has been acquired. Pro-inflammatory ligands activate FPR2 by coupling to Gαi resulting in Ca^2+^ mobilization and ERK phosphorylation, whereas pro-resolving mediators induce β-arrestin 2 recruitment, cAMP production and p38 MAPK phosphorylation. β-arrestins probably mediates heterologous desensitization of FPR2. Subsequent FPR2 internalization is essential to pro-resolving ligands as the internalized receptor inhibits NF-κB (59). In neutrophils, the recruitment of β-arrestin is crucial for FPR2 reactivation and cell migration, as demonstrated by the cell stimulation with conventional FPR2 agonist WKYMVM (60).

On the note, recent studies indicated that FPR2 expression could be regulated by a combination of hormones, genetic, and epigenetic factors. Sex-specific differences of FPR2 expression have been observed in mice. Particularly, estrogen directly regulates FPR2 expression as a protective mechanism against nonalcoholic fatty liver disease (61). In pancreatic tumor, FPR2 exerts immunosuppressive function in females, suggesting a potential sex‐specific approach for immunotherapy (62). Accumulating evidence showed that FPR2 expression could be regulated by microRNAs, especially during inflammation resolution. Pierdomenico AM et al. demonstrated that miR-181b directly binds FPR2 3′-UTR and controls pro-resolution signals (63). Epigenetic regulation of FPR2 promoter and heritable genetic variants that impair promoter activity have been discovered (64). The promoter polymorphism rs11666254 downregulates FPR2/ALX expression and increases risk of sepsis (65). Overall, the regulatory mechanisms of FPR2 expression should be investigated in future studies to provide innovative approaches to inflammatory diseases.

### FPR3 is the least known member of the FPR family

FPR3 still remains poorly characterized. Unlike FPR1 and FPR2, FPR3 does not serve as receptor for fMLF, which represents the established peptide released at sites of bacterial infections and tissue injury. It shares 56% and 83% of its amino acid sequence with FPR1 and FPR2, respectively. FPR3-expressing cells are mainly monocytes/macrophages and endothelial cells (66).

The structure of FPR3 has been largely unknown, although some experimental techniques have been suggested for performing structural analysis. For example, T4-lysozyme (T4L) fusion in the intracellular loop of a G protein-coupled receptor (GPCR) of FPR3 were expressed in stable tetracycline-inducible HEK293 cells and guaranteed approximately 0.2 mg of highly purified monomeric human FPR3-T4L from 2 g of cells. Since T4L fusion did not disturb the proper folding and functionality of FPR3, this strategy could facilitate ongoing efforts in the structural analysis of FPR3 (67).

Of note, FPR3 binds non-formylated peptides. ANXA1, which mediates the anti-inflammatory effects of glucocorticoids, is the first endogenous ligand of FPR3 (68). However, the high affinity ligand for FPR3 is F2L peptide, an acetylated amino-terminal peptide of the human heme-binding protein. In FPR3-expressing leucocytes, F2L induces calcium mobilization and chemotaxis. F2L thereby may be considered as a pro-inflammatory stimulus for FPR3. It has been demonstrated that F2L is generated after tissue damage and induces the recruitment of macrophages and dendritic cells, which contribute to tissue repair and resolution of inflammation (69). This data suggests a pro-resolutive action of FPR3. Another agonist for FPR3 is humanin, a mitochondrial-derived peptide with cytoprotective effects in many cell types. Humanin does not bind exclusively FPR3, but also FPR2 (70).

Interestingly, FPR3 presents a significant basal level of phosphorylation in the absence of ligand, probably due to the presence of Pro^329^. The clathrin-pathway is required for its constitutive internalization (71). Corroborating data demonstrated that FPR3 is expressed in small intracellular vesicles, unlike FPR1 and FPR2 that are expressed on the cell surface. Besides, it has been recently found that FPR1 is expressed intracellularly, while FPR1 and FPR3 were expressed in the nuclei of naïve CD4 T cells. Intracellular and/or nuclear FPR1 but not FPR3 induced chemotactic migration of naïve CD4 T cells (72).

In 2011, Rabiet MJ proposed that the constitutive internalization of FPR3 may serve to regulate the function of FPR1 and FPR2, but these hypotheses have never been confirmed (71).

From data present in literature, it emerges that FPR3 prevalently exerts anti-inflammatory and pro-resolutive actions, as demonstrated by the binding to regulatory peptide against inflammatory processes. Moreover, the intracellular localization of FPR3 opens new questions about the role of FPR family and the identification of endogenous ligands for intracellular FPR members could clarify their functional roles in inflammation and tissue repair. It is conceivable that the lack of scientific data on FPR3 could be linked to the absence of known ligands for intracellular and extracellular FPR3. For example, a recent analysis of the effects of d−Peptide analogues of Boc−Phe−Leu−Phe−Leu−Phe−COOH (used extensively as a FPR1/FPR2 antagonist) on neovascularization showed that this peptide elicited the angiogenic activity via FPR3, which is expressed by endothelial cells (73).

## FPR members are molecular targets for diagnosis and treatment of human diseases

Based on their expression on a wide variety of cell types and their ability to bind ligands with diverse structural and functional features, FPRs have been implicated in multiple human diseases. In particular, the contribution of FPRs to inflammation and cancer places these receptors in the list of molecular targets for new therapeutic approaches. In addition, the study of the functional roles of FPRs in the mucosal immune system has attracted great interest. Mucosal immune cells, including innate lymphoid cells, epithelial cells, and intraepithelial lymphocytes, express FPRs that contribute to maintaining tissue homeostasis at these sites. Recent research focuses on the controversial role of FPRs in the mucosal system depending on their ligands expressed under various pathophysiological conditions (74). Although many reports indicate that FPRs are crucial for maintaining epithelial barrier integrity, a link between FPRs and inflammatory conditions affecting mucosal system, especially gastrointestinal tract and respiratory system, have been found, as described below.

### FPR1 is a critical component of tumor microenvironment

Given that FPR1 plays a crucial role in the inflammatory reactions implicated in disease pathogenesis, FPR1 antagonists represent a promising therapeutic approach in inflammation and cancer.

Zhou et al. were among the first to recognize the involvement of FPR1 in tumorigenesis, demonstrating that FPR1 is expressed by highly malignant human glioma cells and mediate motility, growth, and angiogenesis of human glioblastoma (74). The mechanism implicated in FPR1-mediated glioma growth has been elucidated. ANXA1 released from necrotic tumor cells induces the growth of glioblastoma via the activation of FPR1 (75). Later, Zheng et al. demonstrated that ANXA1 was mainly expressed in cancer cells from high-grade glioma (HGG), whereas FPR1 was predominantly expressed in macrophages and microglia, suggesting that cancer cells interact with macrophages and microglia through the ANXA1/FPR1 axis to inhibit the immune response against glioma (76). These data reveal a new perspective of immunotherapy for glioma, which is characterized by a highly immunosuppressive tumor microenvironment.

A pro-metastatic role in ovarian cancer has been assigned to FPR1. uPAR triggers intra-abdominal dissemination of epithelial ovarian cancer cells through the interaction of its 84-95 sequence with the FPR1. The inhibition of this interaction by anti-uPAR_84-95_ Abs or RI-3 peptide significantly decreased the extent of cell adhesion (41). Elevated levels of FPR1 were associated with poor prognosis in cervical cancer patients (77). The over-expression of FPR1 has also been associated with drug-resistance in bladder cancer (78).

In contrast to the data described above, Prevete et al. demonstrated a protective role of FPR1 in tumor growth and progression. In gastric cancer, FPR1 suppresses tumor growth and angiogenesis due to a pro-resolving action (79). In the same way, the commensal bacterium *Lactobacillus rhamnosus* GG (LGG) exerts pro-resolving, antiangiogenic and homeostatic functions by activating FPR1 in colorectal carcinoma cells (80).

In other studies, the interaction between ANXA1 and FPR1 appears to be relevant in immune response against dying cancer cells. Of interest, anthracyclines-based chemotherapy didn’t exert therapeutic effects in FPR1 knockout tumor-bearing mice as Fpr1-deficient dendritic cells could not elicit T cell immunity against dying tumor cells (81).

The role of FPR1 in immunity and inflammation has been considered “ambiguous” (42). In infectious diseases, FPR1 plays an ambivalent role. In *Escherichia coli* and *Listeria monocytogenes*, FPR1 activation is crucial for host defense, as demonstrated in mouse strains deficient in Fpr1 (82, 83). Conversely, the absence of FPR1 provides protection against *Yersinia pestis*, which is the causative agent of plague (84). In COVID-19, FPR1 signaling is linked to pulmonary fibrosis and other long-term symptoms by inducing excessive alarmins S100A8/A9 (85).

FPR1 triggered the spread of inflammation, cardiomyocyte apoptosis and ventricular remodeling through a positive regulation of MAPK signaling pathway, as demonstrated in animal models of ischemia/reperfusion injury (86). In ischemic retinopathy, FPR1 contributed to inflammation and hindered reparative angiogenesis, thus implying its involvement in neuroretinal dysfunction (87).

In chronic inflammatory disease, there are contrasting data on the role of FPR1. In collagen‐induced arthritis, FPR1 induces beneficial effects by contributing to the inhibitory effects on TH1 and TH17 cell generation, which are crucial for disease progression (88). Conversely, other studies assign a pathogenetic role to FPR1 in diseases conditions associated with dysregulated inflammation, such as celiac disease, cigarette smoke-induced airway inflammation disease, and inflammatory bowel disease (89–91). For example, gliadin, a food antigen that binds to FPR1, decreases intestinal integrity and stimulates neutrophil migration (89). As demonstrated in the intestinal tract, FPR1 plays a crucial role also in inflammation-related diseases of respiratory system. FPR1 is overexpressed in neutrophils isolated from smokers, as compared to non-smokers. Since smoking cessation cannot revert lung inflammation, the FPR blockade prevents structural deterioration of the lungs in mice after smoking cessation (92).

These findings demonstrate the importance of FPR1-mediated neutrophilic chemotaxis that drives the persistence of inflammation and affects mucosal homeostasis in the gastrointestinal and respiratory tracts. As a result, FPR1-targeted therapy could be a novel direction for inflammatory diseases. Further studies could be extended to other mucosal surfaces, such as the oral cavity, the eye, and the reproductive tract.

Of interest, the gene encoding FPR1 is highly polymorphic and numerous single nucleotide polymorphisms (SNPs) have been positively or negatively linked to various diseases.

Variation affecting intracellular domain of FPR1 has been associated with failure of anti-cancer immunotherapies, the age at which the disease manifests, as well as negative prognosis. The loss-of-function provoked by rs867228 is associated with poor responses to anthracycline-based adjuvant chemotherapy in breast cancer and colorectal cancer patients (81). Epidemiological studies showed that both homo- and heterozygosity for rs867228 in FPR1 accelerates age at diagnosis of luminal B breast cancer by 4.9 years (93). rs867228 homozygosity is associated with anticipated diagnosis in other cancer types, such as esophageal carcinoma, head and neck and colorectal cancer (94).

Recently, it has been proposed that the most abundant SNPs in FPR1 may have been selected to enhance human survival against infectious diseases. The most fitting example is represented by rs5030880 that affects the extracellular loop of FPR1 and is considered as a human resistance allele that protects neutrophils from *Yersinia pestis* type III secretion system (T3SS), which selectively destroys human immune cells (84).

### FPR2 as a valid target for “resolution pharmacology”

In the last decade, the complex network of mediators and pathways triggering the resolution of inflammation has been proposed as innovative therapeutic approaches for chronic diseases. In this perspective, FPR2 has been recently defined as a prototype to kick-start “resolution pharmacology” (95).

FPR2 pro-resolving agonists, including lipid mediators such as LXA4, RvD1, and ANXA1, have been shown to exert therapeutic potential in diverse experimental models of chronic diseases. For example, the outcome of Influenza A, caused by a virus from the *Orthomixoviridae* family, improved with LXA4 treatment in mice, by decreasing cell recruitment and pro-inflammatory cytokines release (96). In diabetic mice, RvD1 promoted corneal epithelial wound healing and the restoration of mechanical sensation (97). ANXA1 exerted a therapeutic potential in diabetes and microvascular disease, by protecting peripheral organs against the injury and dysfunction caused by hyperglycemia and hyperlipidemia (98).

To develop new FPR2-agonist biased compounds more resistant to degradation than natural peptides, different techniques and strategies have been adopted by drug design and discovery.

Among synthetic LXA4 mimetics, 6C-dimethyl-imidazole (1R)-11 was found to be the most potent and efficient anti-inflammatory agent by inhibiting peritonitis-associated neutrophil infiltration *in vivo* (99). Compound 17b, a small-molecule ANXA1 mimetic, mediated aorta vasodilation, reduced vascular remodeling associated with myocardial infarction and diabetes, and exerted anti-inflammatory action in pulmonary arterial hypertension (100, 101). Compound MR-39, an FPR2 ureidopropanamide agonist, alleviated the inflammatory process in mouse model of Alzheimer’s Disease (102).

An interesting field of investigation with ongoing clinical trials focused on cardiovascular disorders. Compound 43, a dual agonist of FPR1/2, preserved cardiac structure and function after myocardial infarction by stimulating pro-resolution macrophages and improving left ventricle and infarct structure (103). Two small molecule FPR2 agonists, ACT-389949 (NCT02099071, NCT02099201) and BMS-986235 (NCT03335553), have been investigated in phase 1 clinical trials, though the results on therapeutic efficacy have not been still published (104). ACT-389949 was shown to be safe and well tolerated in healthy subjects. BM986235 was shown to contribute to the resolution of cardiac inflammation and improve both cardiac structure and function post myocardial infarction in animal models. Interestingly, ACT-389949 induces a potent desensitization of FPR2, placing limits on the chronic administration of this compound in cardiovascular disorders (105).

Additionally, a phase 1 clinical trial to analyze the safety and preliminary efficacy of an LXA4 analog, methyl ester-benzo-lipoxin A4 (BLXA4), in patients with gingival inflammation was performed. BLXA4 treatment reduced local inflammation and increased the levels of pro-resolution molecules (106).

FPR2 agonists could be used as therapeutics in inflammatory diseases, but further studies on biased FPR2/ALX agonism towards pro-resolution are needed.

### FPR3 is a key immune-related regulator of tumor microenvironment

The involvement of FPR3 in human disease is still poorly understood. However, diverse groups of research have focused on the role of FPR3 in the regulation of tumor microenvironment. In breast cancer, FPR3 was found to be a prognostic marker in disease progression and individual survival, being significantly associated with FPR3 expression (107). In addition, a tumor suppressor role was assigned to over-expressed FPR3 in gastric cancer cells, by interfering with glycolytic metabolism, proliferation and stemness of tumor cells (108).

A different pathogenetic and clinical relevance of FPR3 was found in glioma, in which FPR3 expression levels were related to the infiltration of immune cells, affecting the glioma immune microenvironment. In fact, the author found that FPR3 correlated positively with immune checkpoint gene PD-1, which is an important immune checkpoint target in glioma immunotherapy (109). Additionally, FPR3 was found to be involved in the control of immune cells and immune response, as demonstrated by a greater abundance of PD-L1 in the gliomas with high-expression levels of FPR3. More interestingly, CD163+FPR+ macrophage subsets were identified by single-cell RNA sequencing in glioma patients and a prognostic model based on the risk score of these cell subsets were constructed using machine learning (110). In head and neck squamous cell carcinoma, a single-cell transcriptomic analysis of T_reg_ demonstrated a significant decrease in ANXA1/FPR3 pair (111).

Whether FPR3 plays a negative or positive role in cancer progression is still debated. However, the emerging evidence described above indicates that FPR3 expression profile has clinical relevance in terms of tumor immunity assessment. All together, these findings indicate that FPR3 can be employed as a promising therapeutic target and/or prognostic indicator both for its ability to modulate glycolysis and stemness of cancer cells and its involvement in tumor immune escape. If future studies confirm that FPR3 is an immune-related biomarker predicting a poor prognosis for cancer, it could be useful for the management of immunotherapy protocols.

In inflammation-associated disease, the role of FPR3 is still uncertain. In a mouse sepsis model, the administration of a selective FPR3 pepducin agonist, which specifically targets the intracellular loops of G protein-coupled receptors, inhibited lung injury, splenocyte apoptosis, and inflammatory cytokine production, significantly increasing the survival rate (112). In systemic sclerosis, the overexpression of FPR3 was confirmed both at protein and mRNA levels in skin fibroblasts from 10 patients. These data agreed with the *in vivo* observations by histological analysis (9). Additionally, FPR3 emerged in the top 10 hub genes involved in cutaneous lupus erythematosus by bioinformatics analyses of gene expression profile (113). It has recently been proposed that FPR3 expressed in human dendritic cells might mediate allergic T_H_2 immune responses. Peptides derived from the allergenic lipocalins but not for peptides derived from the nonallergenic homologues might be binding partners of FPR3, suggesting an involvement of FPR3 in allergic responses to lipocalin allergens (114).

## Summary and concluding remarks

During the past decade, considerable progress has been made in understanding of the FPR role in inflammation and its resolution. Although our understanding of the downstream signaling events following FPR remains incomplete, we tried to put the pieces of intricate puzzle together. Therefore, we dissected the pro-inflammatory and pro-tumorigenic signaling from anti-inflammatory and pro-resolving signaling.

**Figure 1:**
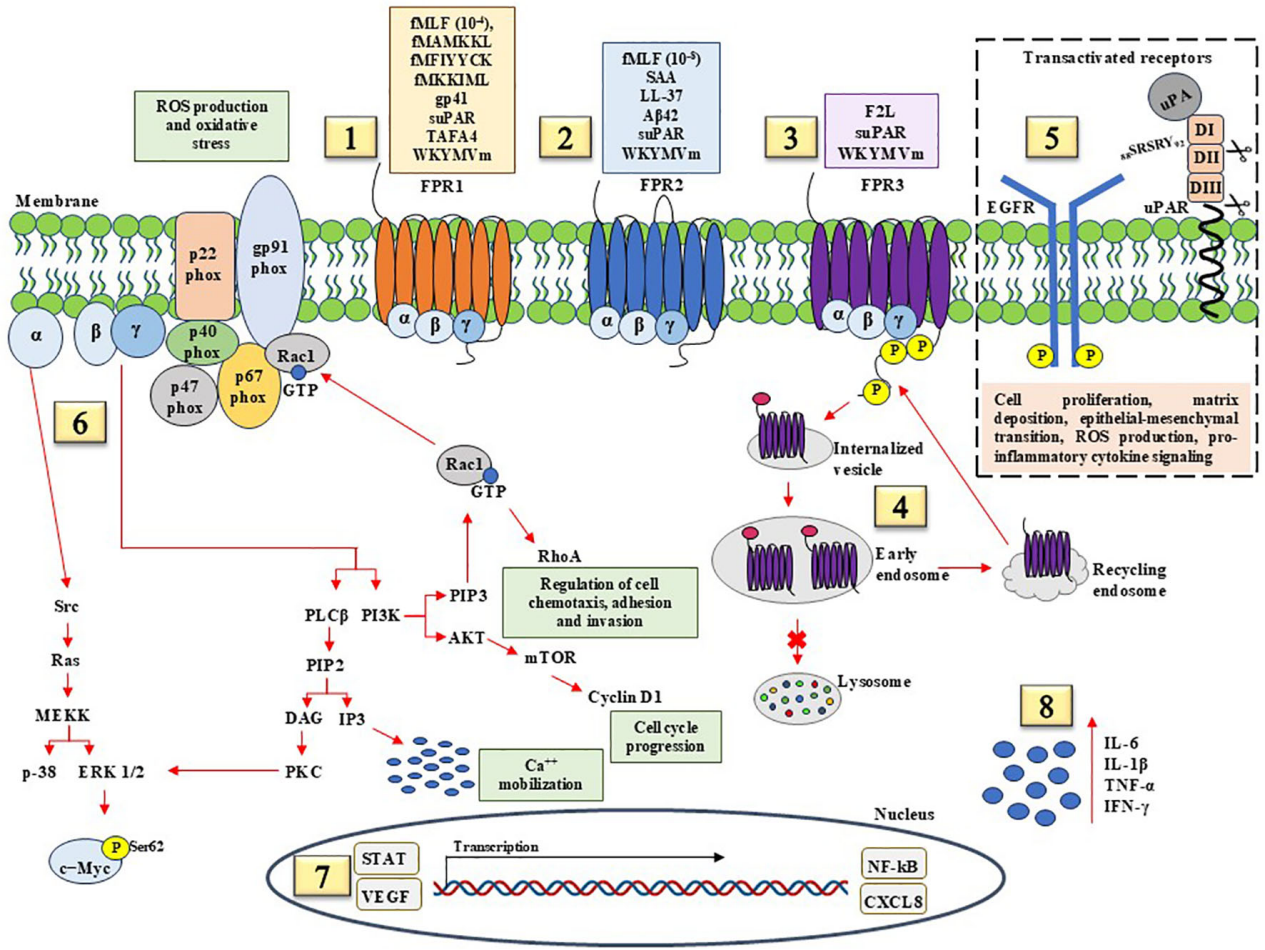
Pro-inflammatory and pro-tumorigenic signaling pathways mediated by FPRs. (1) FPR1 recognize microbe- derived formylated peptides, including *E. Coli*-derived fMLF, *Salmonella*-derived fMAMKKL, *Staphylococcus*-derived fMIYYCK, *Listeria*-derived fMKKIML; HIV-derived non formylated peptides gp41; soluble Urokinase Receptor (suPAR); TAFA chemokine like family member 4 (TAFA4); WKYMVm (Trp-Lys-Tyr-Met-Val-D-Met) synthetic hexapeptide. (2) FPR2 agonists with pro-inflammatory effects include fMLF, Serum Amyloid A (SAA), antimicrobial peptide LL-37, suPAR, and WKYMVm synthetic peptide. (3) FPR3, with significantly basal levels of phosphorylation, binds F2L, a peptide derived from heme-binding protein, suPAR, and WKYMVm synthetic peptide. (4) FPR3 is found mainly distributed in early and late endosome during acute inflammation, suggesting FPR3 continuous recycling following endocytosis in response to pro-inflammatory stimuli. (5) Pro-tumorigenic effects of FPRs are mediated in part by transactivation of epidermal growth factor (EGF) receptor (EGFR) and membrane-anchored uPAR. FPRs/EGFR and FPRs/uPAR crosstalk plays a central role in cell proliferation, matrix deposition, epithelial-mesenchymal transition, ROS production, and pro-inflammatory cytokine signaling. (6) The activation of FPRs from binding pro-inflammatory ligands results in the dissociation of the Gα from the Gβγ subunit. α subunit activates the Ras superfamily, which contribute to activation of the MAPK pathways, p38, and ERK1/2. FPR-mediated ERK activation results in c-Myc Ser62 phosphorylation that has been observed in numerous cancer cells. βγ subunits activate PLCβ, resulting in calcium release from intracellular stores, PKC and PI3K which further contribute to activation of Rac1 GTPase. Rac1 induces the assembly and activation of NADPH oxidase to produce reactive oxygen species (ROS), Rho GTPases to facilitate cancer cell metastasis by regulating actin and proteins associated with cell migration and invasion, and Akt/mTOR axis to enhance cyclin D1 and cell cycle progression. (7) The effects of pro-inflammatory and pro-tumorigenic ligands culminate in the activation of transcription factors including STAT, VEGF, NF-κB, and CXCL8. (8) Pro-inflammatory agonist peptides promote the production of pro-inflammatory cytokines, including IL-6, IL-1β, TNF-α, and IFN-γ.

Pro-inflammatory and pro-tumorigenic signals by FPRs contribute to the activation and recruitment of immune cells and the assembly of NADPH oxidase, thereby promoting cell chemotaxis, calcium mobilization, and ROS production. Biochemical pathways involved in these activities are reported in **Figure 1**. Well-consolidated pro-inflammatory ligands are specific for FPR1, FPR2, and FPR3 or can stimulate all members of FPR family, such as WKYMVm synthetic peptide that is considered as a pan-agonist for FPRs. It is conceivable that FPR3, characterized by significant basal levels of phosphorylation, continuously recycles across the membrane when stimulated by pro-inflammatory ligands, having been found in early and late endosomes. FPRs can be involved in macromolecular complexes on cell surface, by the transactivation of EFGR and uPAR, thus enhancing cell proliferation, matrix deposition, epithelial-mesenchymal transition, ROS production, and pro-inflammatory cytokine release. Upon binding of agonists, FPRs activate heterotrimeric G proteins, which dissociates into α and βγ subunits. Intracellular effectors of α subunits include the mitogen-activated protein kinases ERK1/2 and p38, whereas βγ signaling activates PLC. ERK 1/2 could be involved in Myc protein stability by phosphorylation at serine 62 (Ser62) residue, which is crucial for cell cycle progression and cell proliferation. Hydrolysis of phosphatidylinositol 4,5-biphosphate (PIP2) by PLC generates IP3, which induces the release of calcium from endoplasmic reticulum stores, and diacylglycerol (DAG), which activates PKC isoforms. The phosphoinositide 3-kinase (PI3K)/Akt/mTOR pathway is also activated, thus inducing an increase in cyclin D1 expression and cell cycle progression from G_0_/G_1_ to S phase. Together, these findings support the existence of an NF-κB-mediated pathway and the activation of proangiogenic and inflammatory transcription factors including STAT, VEGF, and CXCL8. These events are accompanied by the release of pro-inflammatory cytokines, including IL-6, IL-1β, TNF-α, and IFN-γ.

**Figure 2:**
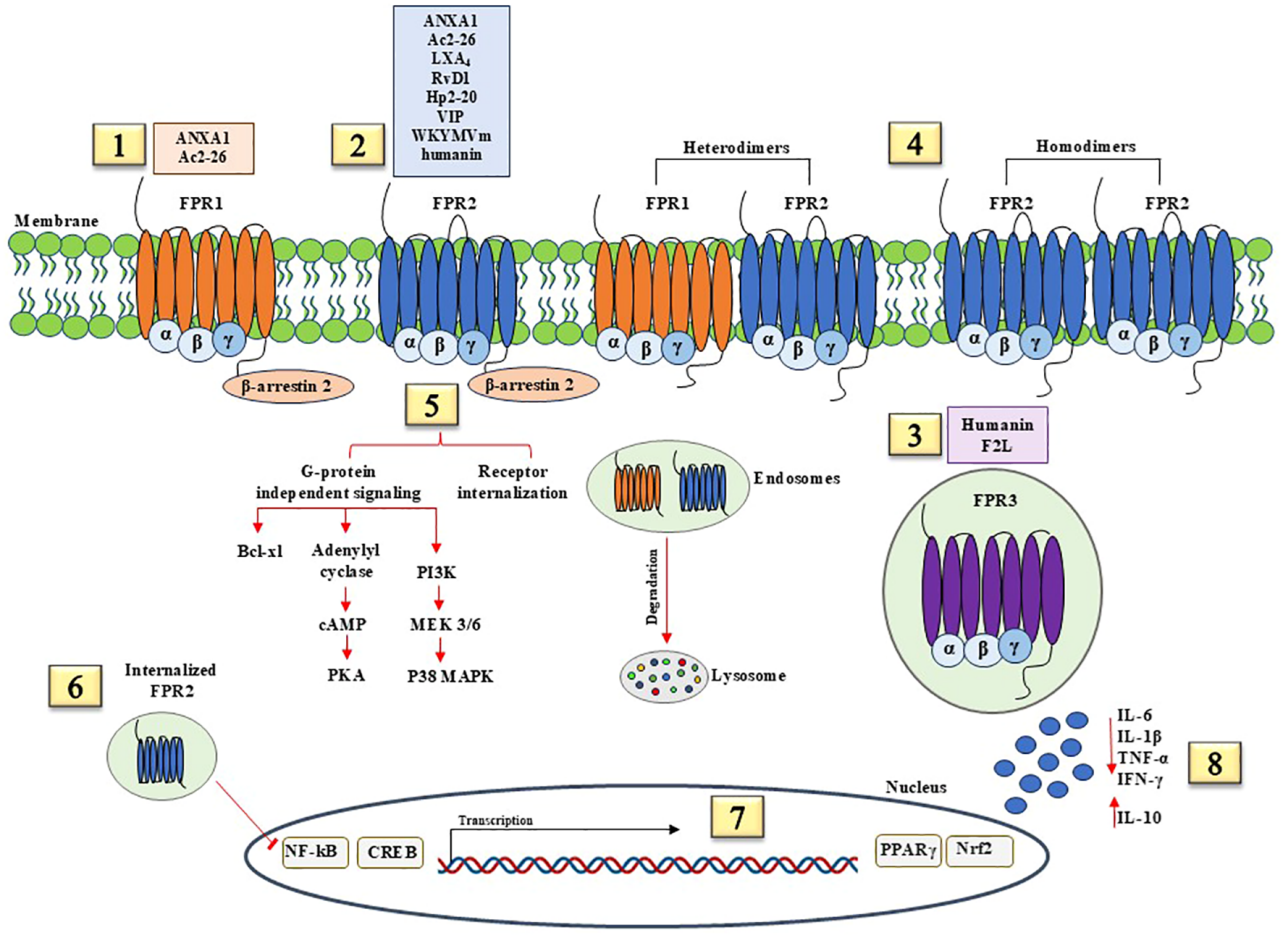
Anti-inflammatory and pro-resolving signaling pathways mediated by FPRs. (1) FPR1 agonists with anti-inflammatory effects include Annexin A1 (ANXA1) and N-terminal peptide of ANXA1, Ac2-26. (2) FPR1 agonists with anti-inflammatory effects include ANXA1, Ac2-26, lipid mediators Lipoxin A4 (LXA4) and Resolvin D1 (RvD1), Hp2-20 peptide of *Helicobacter pylori*, Vasoactive intestinal peptide (VIP), WKYMVm synthetic peptide, and humanin, a recently identified neuroprotective factor. (3) FPR3 is constitutively internalized and binds humanin and F2L. (4) FPR1/FPR2 heterodimers and FPR2 homodimers elicit pro-resolving and pro-inflammatory effects. (5) Pro-resolution and anti-inflammatory pathways start with β-arrestin 2 recruitment, which induces receptor desensitization and internalization and G-protein independent signaling. β-arrestin 2 promote bcl-xL expression, leading to cell survival, cAMP-mediated signaling, and p38 MAPK phosphorylation. (6) FPR2 internalization is crucial for the resolution of inflammation as the internalized receptor inhibits NF-κB activity. (7) The activation of anti-inflammatory and pro-resolving pathways mediated by FPRs induces the transcription of cAMP response element-binding protein (CREB), peroxisome proliferator-activated receptor (PPARγ), and nuclear factor erythroid 2–related factor 2 (NRF2). (8) FPR pro-resolving agonists upregulate anti-inflammatory factors, including IL-10, and block the release of pro-inflammatory cytokines, such as IL-6, IL-1β, TNF-α, and IFN-γ.

On the other hand, anti-inflammatory and pro-resolving signals by FPRs contribute to activating and coordinating processes aimed at restoration of tissue integrity and function, as shown in **Figure 2**. FPR ligands with pro-resolving effects may cause receptor homodimerization or heterodimerization with other FPRs. FPR3 is constitutively internalized and can bind anti-inflammatory ligands. Pro-resolution and anti-inflammatory pathways start with β-arrestin 2 recruitment, which induces receptor desensitization and internalization and G-protein independent signaling. How this determinate the shift from pro-inflammatory to anti-inflammatory functions of FPRs and how FPR signaling pathways bifurcate into canonical and non-canonical transduction are still under investigation. Probably, biased allosteric modulators induce conformational changes in FPRs and generate pro- or anti-inflammatory signals, as demonstrated for FPR2. Specifically, FPR2 has two allosteric binding sites, each for one specific ligand, with pro- or anti-inflammatory activity. The recruitment of β-arrestin 2 to cytoplasmic portion of FPR2 is induced by anti-inflammatory ligands, whereas pro-inflammatory ligands elicit the release of calcium from endoplasmic reticulum stores and ERK phosphorylation (26). β-arrestin 2 promotes bcl-xL expression, leading to cell survival, cAMP-mediated signaling, and p38 MAPK phosphorylation. The internalization of FPR2 is crucial for the resolution of inflammation as the internalized receptor inhibits NF-κB activity. The activation of anti-inflammatory and pro-resolving pathways mediated by FPRs induces the transcription of cAMP response element-binding protein (CREB) that promotes anti-inflammatory responses through the inhibition of NF-κB activity and the induction of IL-10, peroxisome proliferator-activated receptor (PPARγ) that represses the NF-κB pathway, NRF2 that protects against oxidative damage. FPR pro-resolving agonists upregulate anti-inflammatory factors, including IL-10, and decrease pro-inflammatory cytokine production.

Finally, uncontrolled and unresolved inflammation may become chronic and lead to a pathological disease state; in this context, the modulation of FPR signaling could be instrumental in resolving pathologic inflammation. Particularly attractive are the immunomodulatory role of FPR2 by regulating proinflammatory and anti-inflammatory activities and the association of FPR3 with tumor immunity, indicating its availability as a prognostic biomarker in cancer. A better understanding of FPR signaling is necessary to aid the drug development process, in order to stimulate the desirable biological properties.
